# Design and Implementation of a Micromechanical Silicon Resonant Accelerometer

**DOI:** 10.3390/s131115785

**Published:** 2013-11-19

**Authors:** Libin Huang, Hui Yang, Yang Gao, Liye Zhao, Jinxing Liang

**Affiliations:** 1 Key Laboratory of Micro-Inertial Instrument and Advanced Navigation Technology, Ministry of Education, Nanjing 210096, China; E-Mails: huanglibin@seu.edu.cn (L.H.); yhtriangle@foxmail.com (H.Y.); gaoyang881120@126.com (Y.G.); j-liang@seu.edu.cn (J.L.); 2 School of Instrument Science and Engineering, Southeast University, Nanjing 210096, China

**Keywords:** accelerometer, MEMS, resonant, temperature characteristic, structure design, drive circuit

## Abstract

The micromechanical silicon resonant accelerometer has attracted considerable attention in the research and development of high-precision MEMS accelerometers because of its output of quasi-digital signals, high sensitivity, high resolution, wide dynamic range, anti-interference capacity and good stability. Because of the mismatching thermal expansion coefficients of silicon and glass, the micromechanical silicon resonant accelerometer based on the Silicon on Glass (SOG) technique is deeply affected by the temperature during the fabrication, packaging and use processes. The thermal stress caused by temperature changes directly affects the frequency output of the accelerometer. Based on the working principle of the micromechanical resonant accelerometer, a special accelerometer structure that reduces the temperature influence on the accelerometer is designed. The accelerometer can greatly reduce the thermal stress caused by high temperatures in the process of fabrication and packaging. Currently, the closed-loop drive circuit is devised based on a phase-locked loop. The unloaded resonant frequencies of the prototype of the micromechanical silicon resonant accelerometer are approximately 31.4 kHz and 31.5 kHz. The scale factor is 66.24003 Hz/g. The scale factor stability is 14.886 ppm, the scale factor repeatability is 23 ppm, the bias stability is 23 μg, the bias repeatability is 170 μg, and the bias temperature coefficient is 0.0734 Hz/°C.

## 1. Introduction

A micromechanical silicon resonant accelerometer converts the acceleration signals to be tested into the frequency variation of the resonator. Thus, the output is a quasi-digital signal. Moreover, the micromechanical silicon resonant accelerometer has the advantages of a wide dynamic range, strong anti-interference capacity and high stability. The output signal need not experience A/D conversion before entering the digital system, which greatly facilitates the signal processing. Thus, this type of sensor can easily achieve high-precision measurements. In addition, it possesses the numerous other advantages of silicon micro-inertia devices. It is one of several new-generation, high-precision MEMS accelerometers.

In recent years, the micromechanical silicon resonant accelerometer has invoked great interest worldwide. Some famous companies and research institutions have thoroughly studied this type of accelerometer [1–15]. The micromechanical silicon resonant accelerometer designed by Honeywell is driven by electrostatics and detected using piezoresistance. The initial devices were fabricated with scale factors greater than 700 Hz/g on +20 g. The performed temperature tests indicated frequency shifts of approximately 45 ppm/°C. The short-term stability with respect to the microbeam base frequency is better than 0.1 ppm [1].

A prototype device that was developed by the University of California, Berkeley, has base resonator frequencies of 145 kHz and a scale factor of 17 Hz/g [3]. Sung from Seoul National University developed a type of micromechanical silicon resonant accelerometer that was driven and detected using parallel capacitors, which were tuned by the electrostatic negative stiffness. The unloaded resonant frequency of the resonator is approximately 31.4 kHz. The scale factor is 24.7 Hz/g, and the nonlinearity of scale factor is less than 2%. The bias stability is approximately 0.7 mg, and the dynamic range is over 10 g [8].

Kim from Seoul National University designed inertial-grade vertical-type and lateral-type differential accelerometers. They consist of an out-of-plane (for the z-axis) accelerometer and in-plane (for the x- and y-axes) accelerometers. The sensing principle of the accelerometer is based on the gap-sensitive electrostatic stiffness changing effect. The out-of-plane resonant accelerometer shows a bias stability of 2.5 μg, a sensitivity of 70 Hz/g and a bandwidth of 100 Hz at a resonant frequency of 12 kHz. The in-plane resonant accelerometer shows a bias stability of 5.2 μg, a sensitivity of 128 Hz/g and a bandwidth of 110 Hz at the resonant frequency [10,11].

Draper Laboratory was one of the pioneers in the study of micromechanical accelerometers, and their results remain at the cutting edge of international research. The Draper studies show that a 0.01 °C temperature control will be maintained if the scale factor stability is better than 1 ppm. The principle prototype they developed provides the best overall performance, with a scale factor stability of better than 1 ppm and a bias stability superior to 1 μg [12].

China's research on micromechanical silicon resonant accelerometers started recently. At most institutions, the research remains at the simulation stage of the micromechanical structure. Laboratory prototypes have rarely been developed. In 2004, the Institute of Microelectronics at Peking University, and the Department of Precision Instruments at Tsinghua University, collaboratively developed a prototype of a micromechanical silicon resonant accelerometer with a sensitivity of 27.3 Hz/g and a resolution of 167.8 μg [16]. The micromechanical silicon resonant accelerometer prototype developed by Chongqing University in 2010 has a sensitivity of approximately 55.03 Hz/g and a resolution of approximately 182 μg [17]. Nanjing University of Science and Technology studied the temperature influence mechanism of the micromechanical silicon resonant accelerometer. An improved structure restraining thermal stress and a temperature compensation measure based on electrostatic stiffness was proposed. After the accelerometer is electrically pre-heated for 10 min, the bias stability of the prototype is 100 μg, and the bias repeatability is 286 μg. The scale factor stability is 51 ppm, and the scale factor repeatability is 2.7335 × 10^−3^. The temperature coefficient of the resonator is 42 Hz/°C [18]. The China Academy of Aerospace Electronics Technology launched a micromechanical silicon resonant accelerometer prototype in 2013 with an unloaded resonant frequency of approximately 17 kHz and a scale factor of approximately 220 Hz/g. In the range of −40–+70 °C, the temperature coefficient of the resonant frequency is −71.5 × 10^−6^/°C. The bias stability approaches 42.5 μg within 1.5 h [19].

The domestic SOG technique is currently adopted by most research institutions for fabricating micromechanical silicon resonant accelerometers. In this technique, the anodic bonding process is used to form a tight silicon-oxygen bond to adhere the silicon wafer and the glass wafer together. Because of the mismatching thermal expansion coefficients of silicon and glass, thermal stress will be produced during the fabrication, packaging and use of the accelerometer. This thermal stress will seriously affect the accelerometer performance. In this study, the structure of the micromechanical silicon resonant accelerometer is optimized to reduce the temperature influence on the accelerometer. Thus, the closed-loop drive circuit is designed based on the phase-locked loop. A performance test is also performed on the developed prototype.

## 2. Structural Design of Micromechanical Silicon Resonant Accelerometer

**Figure 1.**
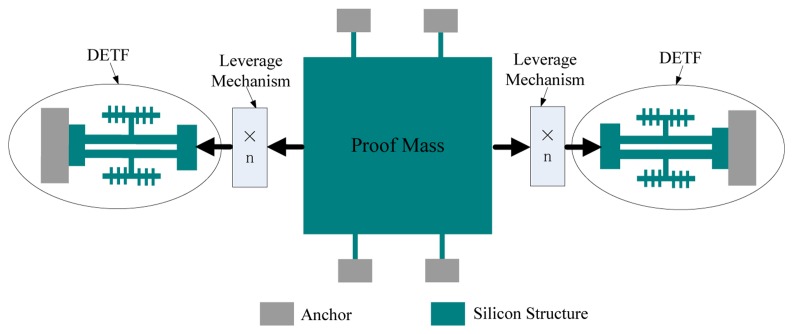
Structural diagram of the micromechanical silicon resonant accelerometer.

A structural diagram of the micromechanical silicon resonant accelerometer is shown in Figure 1. The accelerometer is designed with a perfectly symmetrical differential structure.

Two identical double-ended tuning forks (DETFs) serve as the stress-sensitive resonators. The two DETFs are symmetrically arranged and connected by the proof mass, which converts the acceleration into an inertial force, which is later magnified by leverage before being transmitted to the resonators. The resonant frequency of one resonator will decrease under the compressive force, and the resonant frequency of the other resonator will increase under the tensile force. The magnitude of the input acceleration will be calculated from the difference between the resonant frequencies of the two resonators.

By simplifying the relevant theoretical formula [20], the frequency outputs of the resonators of the micromechanical silicon resonant accelerometer are given as follows:

(1) $$f=f_{0}\sqrt{1\pm F\frac{0.295L^{2}}{Ehw^{3}}}$$

where ± denote the tensile force and the compressive force on the resonators, respectively, *F* is the magnitude of the axial force, *f_0_* is the unloaded resonant frequency of the resonator, *E* is the elastic modulus, *h* is the thickness of the resonant beam, *L* is the length of the resonant beam, and *w* is the width of the resonant beam.

The differential output of the accelerometer is given as follows:

(2) $$\Delta f=f_{0}(\sqrt{1+F\frac{0.295L^{2}}{Ehw^{3}}-}\sqrt{1-F\frac{0.295L^{2}}{Ehw^{3}})}$$

Taylor expansion is performed on Equation (2) with the high-order terms omitted; thus:

(3) $$\Delta f=f_{0}\cdot F\frac{0.295L^{2}}{Ehw^{3}}+\frac{1}{8}f_{0}{\left(F\frac{0.295L^{2}}{Ehw^{3}}\right)}^{3}$$

Equation (3) shows that the differential output that is adopted in the overall design of the accelerometer can provide the following benefits:

- Because the beat frequency is far below the unloaded resonant frequency, the accelerometer bias is greatly reduced.
- The scale factor is two times that of a single resonator.
- The *a^2^* term of the beat frequency is zero (*a* is the input acceleration), which greatly reduces the nonlinearity.
- The effect of the common-mode errors, such as temperature and stress, on the output is weakened.

The DETFs serve as resonators in the micromechanical silicon resonant accelerometer. When there is an acceleration input, the axial force on the resonant beam will induce changes in the resonant frequency. In addition, the thermal stress caused by variations in the ambient temperature results in the variation of the resonant frequency. Thus, the additional stress induced by variations in the ambient temperature should be minimized in the structural design.

**Figure 2.**
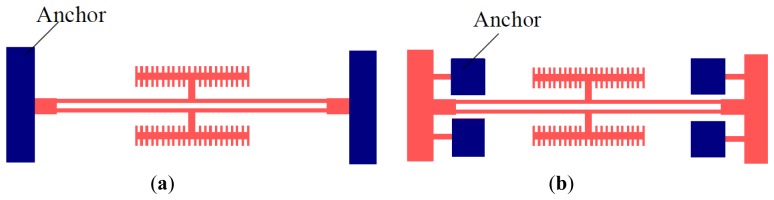
(**a**) The common DETF structure; (**b**) The improved DETF structure.

Figure 2 shows a common DETF structure and the improved DETF structure, respectively. The two schemes share identical resonant beam structures. The only difference is the means of fixation of the anchor. The second design scheme allows for a better release of the axial thermal stress on the resonant beam, which is directly related to the size variation that results from the temperature variation during the structural processing and the operation of the prototype.

**Figure 3.**
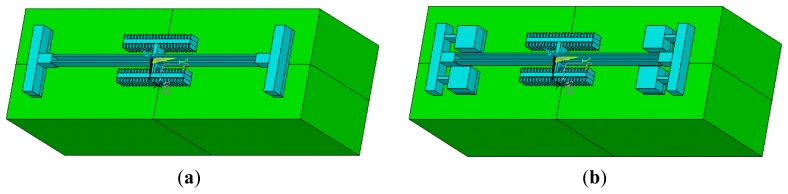
(**a**) Simulation model of the common DETF; (**b**) Simulation model of the improved DETF.

A thermal analysis simulation is performed for the two DETF structures using the ANSYS software. The thermal stress in the DETF structure can be obtained by simulation. Figure 3 shows two types of simulation models for DETF structures. The blue part in the figure is a 60 μm deep silicon layer. The width of the resonant beam is set to 8 μm, and the length is set to 800 μm. The green part in the figure is a 500 μm deep glass layer.

**Figure 4.**
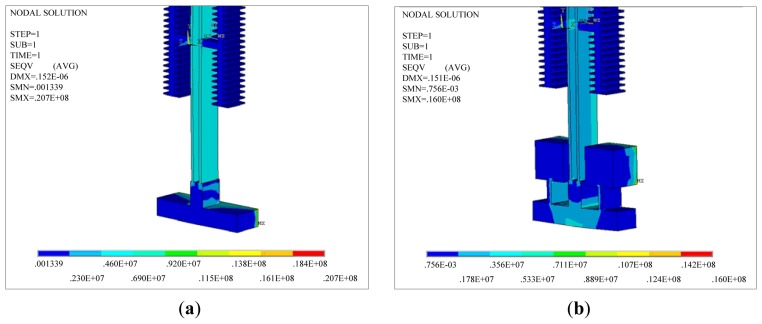
The stress distributions of the two DETF structures when the ambient temperature decreases from room temperature to −40 °C. (**a**) The common DETF structure; (**b**) The improved DETF structure.

**Figure 5.**
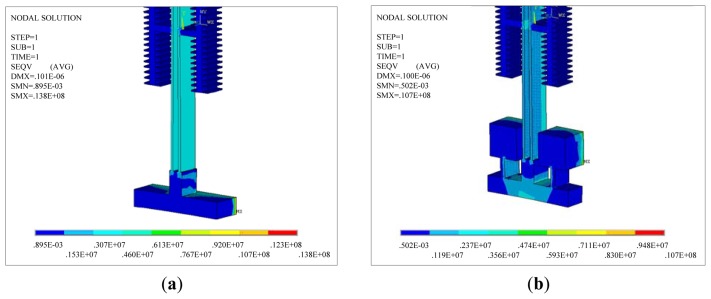
The stress distributions of the two DETF structures when the ambient temperature increases from room temperature to +60 °C; (**a**) The common DETF structure, (**b**) The improved DETF structure.

Figure 4 shows the stress distributions of the two DETF structures when the ambient temperature decreases from room temperature to −40 °C. Figure 5 is the stress distributions of the two DETF structures when the ambient temperature increases from room temperature to +60 °C.

As shown in Figures 4 and 5, the thermal stress on the resonant beam in the improved scheme is smaller than that in the scheme before the improvement (Figure 2a) for an identical ambient temperature variation. The thermal stress is imposed on the structure as pre-stress, and a structural dynamic analysis is performed on the structure. The reverse motion of the two beams is the working mode. The resonant frequency of the reverse motion between the two beams can be obtained at different temperatures as shown in Table 1. Table 1 shows that the improved DETF structure can reduce the influence of thermal stress on the resonant frequency of the resonant beam.

**Figure 6.**
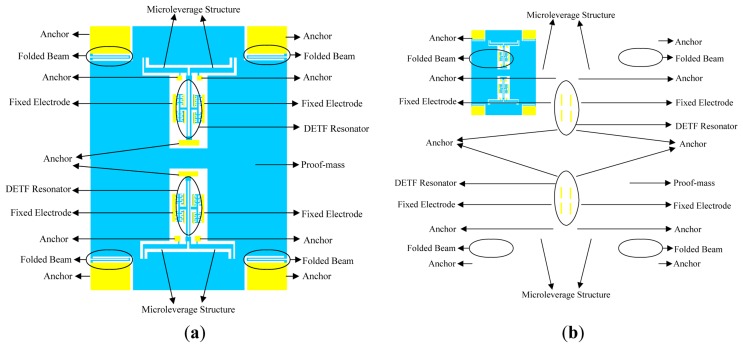
(**a**) The diagram of the accelerometer equipped with the common DETF structure; (**b**) The diagram of the accelerometer equipped with the improved DETF structure.

To amplify the resonant frequency variation, which is caused by acceleration, and to increase the scale factor of the entire device, a single-stage microleverage mechanism is used to magnify the inertial force. The microleverage mechanism is directly connected to the proof mass and the resonators. The major consideration in the structural design is whether the connection mode releases the axial thermal stress on the resonant beam, which is related to the resulting size variation because of the temperature variation. Figure 6 shows diagrams of the accelerometer equipped with the common DETF structure and that equipped with the improved DETF structure, respectively.

**Figure 7.**
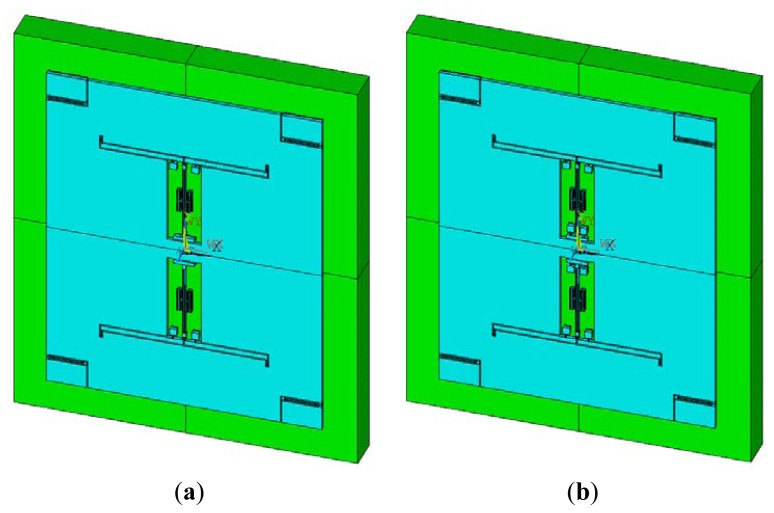
Simulation models of the two types of accelerometers. (**a**) The accelerometer equipped with the common DETF structure; (**b**) The accelerometer equipped with the improved DETF structure.

Figure 7 shows the simulation models of the two types of accelerometers. The parameters of the resonant beam are previously described, and the parameters of the microleverage are shown in Table 2.

A thermal analysis simulation is performed for the two types of accelerometers. The thermal stress caused by the change in the ambient temperature is imposed on the accelerometer as the pre-stress, and a structural dynamic analysis is performed on the accelerometer. The resonant frequency of the reverse motion between the two beams can be obtained at different temperatures, as shown in Table 3. Because the two resonators in the accelerometer are symmetrical, only one resonator is analyzed. Table 3 depicts the resonant frequencies of the upper resonators of the two accelerometers. Table 3 shows that the working frequency shift of the improved DETF is 72.6 Hz, whereas the frequency shift of the common DETF is 331.8 Hz. Obviously, the improved DETF structure can greatly reduce the effect of the thermal stress in the overall accelerometer structure.

Ideally, the frequency shift of the two resonators, which is caused by the change in the ambient temperature, can be eliminated using a differential structure. However, during fabrication and packaging, the highest temperature required by the technology is 400 °C. Because of the mismatching thermal expansion coefficients of silicon and glass, a large thermal stress will be generated in the resonant beams. The large thermal stress may deform or damage the structure.

**Figure 8.**
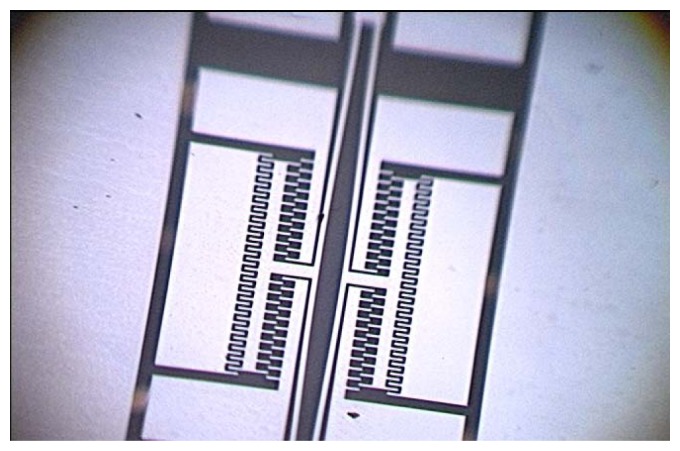
The deformation of the accelerometer with common DETF after packaging.

**Figure 9.**
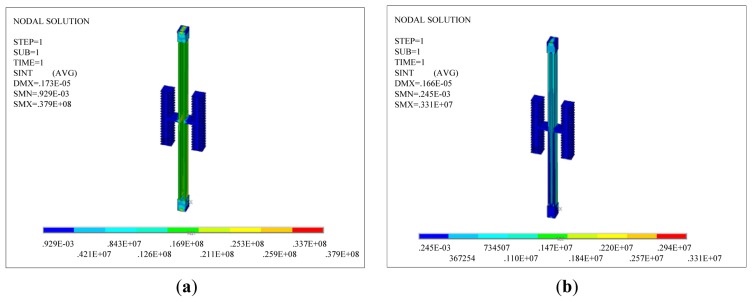
**T**he stress distributions of the two DEFT structures at 400 °C (**a**) The accelerometer equipped with the common DETF structure; (**b**) The accelerometer equipped with the improved DETF structure.

After the accelerometer is cooled from a high temperature to room temperature, there will be a large residual stress in the resonant beams. The residual stress results in a large frequency deviation and may lead to structural deformation or damage. Some accelerometer structures with common DETF have produced deformation after packaging. Figure 8 shows the accelerometer deformation. A thermal analysis simulation is imposed on the two types of accelerometer at 400 °C, and the stress distribution of the resonant beams is shown in Figure 9. The stress in the improved structure is far lower than the old value, which shows that the structure improvement is notably effective.

**Figure 10.**
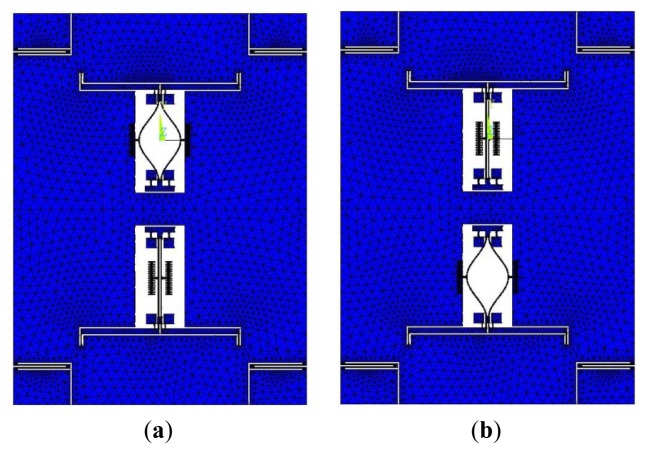
The working modes of the improved accelerometer. (**a**) The working mode of the upper resonator; (**b**) The working mode of the lower resonator.

A structural dynamics simulation is performed on the improved accelerometer, and the working modes are shown in Figure 10.

The working frequencies of the upper resonator and the lower resonator are 31,279.8 Hz and 31,285.6 Hz, respectively. There is a small difference between the two resonators because of the calculation error. When a different acceleration is applied to the accelerometer, a different frequency shift is obtained. The calculated scale factor from the process is 62.6 Hz/g.

## 3. Design of a Closed-Loop Drive Circuit Based on a Phase-Locked Loop

The resonant beam in the micromechanical silicon resonant accelerometer is sensitive to the acceleration variation. In the measuring range, the resonant frequency is allowed to vary over a wide range. In this frequency range, the drive circuit shows an equivalent stable phase shift and accurately tracks the frequency variation of the resonant beam. The phase-locked loop (PLL) is essentially an automatic control closed-loop system that synchronizes the phases of two electric signals. The frequency of the input signals is constantly tracked in a certain range. In addition, this loop strongly inhibits the input noise. When applied to the micromechanical silicon resonant accelerometer, the phase-locked loop has distinctly superior performance [21–24].

### 3.1. Principle of Closed-Loop Driving

**Figure 11.**
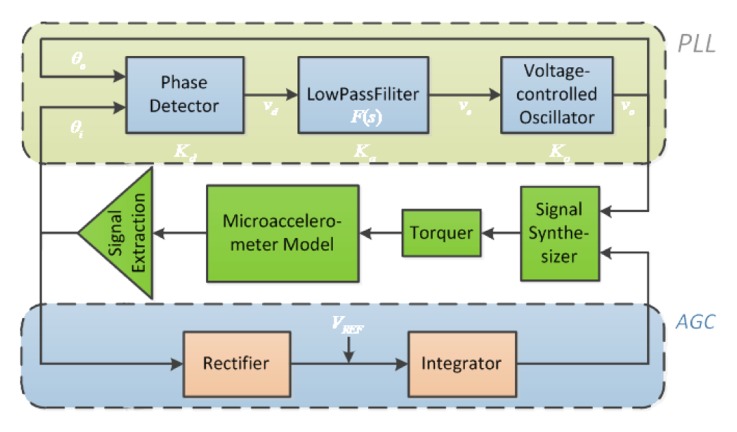
The closed-loop drive circuit diagram of the micromechanical silicon resonant accelerometer.

The closed-loop drive circuit diagram of the micromechanical silicon resonant accelerometer is shown in Figure 11. The upper circuit is used to control the phase using the phase-locked loop (PLL). The lower circuit is used to control the drive amplitude. The integrator averages the amplitude over a time period and produces the gain control coefficient. The resonant frequency of the micromechanical silicon resonant accelerometer varies with the axial acceleration. The phase-locked loop achieves and tracks the frequency through the phase control. Because of the narrowband characteristics, the phase-locked loop can remove most of the noise. The phase-locked loop is equivalent to a filter. The information of the input signal is contained in the phase or the amplitude. The phase-locked loop reconstructs the input signal by using the voltage-controlled oscillator (VCO) [12,23,24].

#### 3.1.1. Phase Control

The dynamics equation of the resonant beam of the micromechanical silicon resonant accelerometer is expressed as follows:

(4) $$M_{n}\ddot{x}+M_{n}\frac{\omega_{n}}{Q_{n}}\dot{x}+K_{n}x=f\cos\theta$$

where *M_n_* is the equivalent mass of the beam, *ω_n_* is the natural angular frequency of the resonant beam, *Q_n_* is the quality factor of the resonator, *K_n_* is the first-order elasticity factor of the beam, *f* is the amplitude of the driving force, and *θ* is the instantaneous phase angle. The instantaneous angular frequency of the voltage-controlled oscillator is [24]:

(5) $$\omega (t)=\omega_{0}+K_{o}v_{e}(t)$$

where *ω*_0_ is the free oscillation frequency of the VOC, *K_o_* is the frequency gain coefficient of the voltage controlled oscillator, and *v_e_(t)* is the control voltage of the voltage-controlled oscillator. The angular frequency refers to the variation rate of the phase angle with time. Thus, the instantaneous phase angle is:

(6) $$\theta_{o}=\int_{0}^{t}[\omega_{0}+K_{o}v_{e}(\tau )]d\tau$$

The open-loop transfer function of the phase-locked loop with respect to the phase is expressed as follows:

(7) $$G(s)=K_{v}\cdot \frac{F(s)}{s}$$

where *K_v_* is the circuit gain, *K_v_* = *K_d_K_a_K_o_*, *K_d_* is the gain coefficient of phase discriminator, *K_a_* is the gain coefficient of the low-pass filter, and *F*(*s*) is the transfer function of the low-pass filter.

The closed-loop transfer function of the system is expressed as follows:

(8) $$H(s)=\frac{\theta_{o}(s)}{\theta_{i}(s)}=\frac{G(s)}{1+G(s)}=\frac{K_{v}F(s)}{s+K_{v}F(s)}$$

where *θ_i_* is the input phase angle of the phase-locked loop, and *θ_o_* is the output phase angle of the phase-locked loop. The error transfer function is deduced as follows:

(9) $$H_{e}(s)=\frac{\theta_{i}(s)-\theta_{o}(s)}{\theta_{i}(s)}=\frac{1}{1+G(s)}=\frac{s}{s+K_{v}F(s)}$$

#### 3.1.2. Amplitude Control

The driving amplitude control circuit is composed of a full-wave rectifier and an integrator. Suppose that the vibration displacement of the resonant beam is written as follows:

(10) x=Asinωt

where *A* is the vibration amplitude. Then, the input voltage of the full-wave rectifier is:

(11) $$V_{1}=K_{S}A\sin\omega t$$

where *K_S_* is the magnification coefficient of the signal pick-up unit. After a full wave rectification, the output voltage is:

(12) $$V_{2}=|V_{1}|$$

The integrator is a low-pass filter. When the cut-off angular frequency is much smaller than *ω*, the output of the integrator is the DC component of the Fourier series of *V_REF_*–*V_2_*, *i.e.*, the mean value:

(13) $${\bar{V}}_{2}=K_{I}\cdot \frac{1}{2\pi }\int_{0}^{2\pi }\left(V_{REF}-|K_{S}A\sin\omega t\right|)d\omega t=K_{I}V_{REF}-\frac{2K_{I}K_{S}A}{\pi }$$

where *K_I_* is the gain of the integrator, and *V_REF_* is the pre-set reference voltage. If *V̄*_2_ is considered the AC drive amplitude, the drive amplitude control constitutes the negative feedback loop of the vibration amplitude, which stabilizes the vibration amplitude of the resonant beam.

### 3.2. Precise 90 Degree Phase Shift

The normalized transfer function of the low-pass loop filter is expressed as follows [21,22]:

(14) $$F(s)=\frac{P(s)}{s^{m}Q(s)}$$

When Equation (14) is substituted into Equation (9), the following expression is obtained:

(15) $$H_{e}(s)=\frac{s^{m+1}Q(s)}{s^{m+1}Q(s)+K_{v}P(s)}$$

The continuous acceleration that slowly varies in a finite time can be approximated as a superimposition of several step-change accelerations as follows:

(16) $$a(t)=\overset{\infty }{\underset{k=1}{\text{∑}}}a(k\Delta )[u(t-k\Delta )-u(t-(k-1)\Delta )]$$

where *a* is the input acceleration, *u*(*t*) is the step-change function, and Δ is the subdivided time interval. The resonant frequency of the resonant beam is linearly related to the acceleration, and the phase is the integral of the angular frequency. For the frequency step-change input *r*(*t*) = *R*·*u*(*t*), where *R* is the step-change amplitude, the steady-state error of the system phase is given by:

(17) $$e_{ss}=\lim_{s\to 0}sH_{e}(s)\cdot \frac{R}{s}=\frac{s^{m}Q(s)\cdot R}{s^{m+1}Q(s)+K_{v}P(s)}$$

In general, the passive low-pass filter has no pole. Therefore, *m* = 0; then:

(18) $$e_{ss}=\frac{RQ(0)}{K_{v}P(0)}$$

If *K_v_* ≫ *ω_n_*, the phase-locked system is a high-gain circuit with a steady-state error of approximately zero. For a large step-change amplitude *R*, the steady-state error should be eliminated to realize the high-precision linear control of the accelerometer.

**Figure 12.**
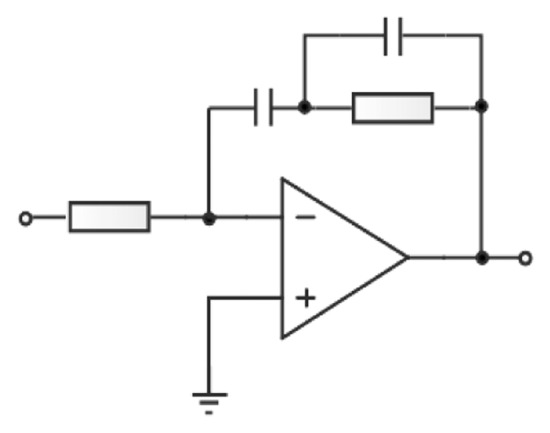
The active proportion-integration filter.

The active proportion-integration filter in Figure 12 is used, which indicates that the integration procedure for the circuit will eliminate the steady-state error and realize the precise 90 degree phase shift.

The transfer function of this procedure is expressed as:

(19) $$F(s)=\frac{1+sT_{2}}{sT_{1}(1+sT_{3})}$$

where *T*_1_=1/*R*_1_*C*_1_, *T*_2_=1/*R*_2_*C*_1_, and *T*_3_=1/*R*_2_*C*_2_. Then, the steady-state error is calculated as:

(20) $$e_{ss}=\lim_{s\to 0}\frac{s^{2}T_{1}T_{3}+sT_{1}}{s^{3}T_{3}+s^{2}T_{1}+sK_{v}T_{2}+K_{v}}=0$$

Thus, the no-error phase control is achieved in the resonant circuit.

**Figure 13.**
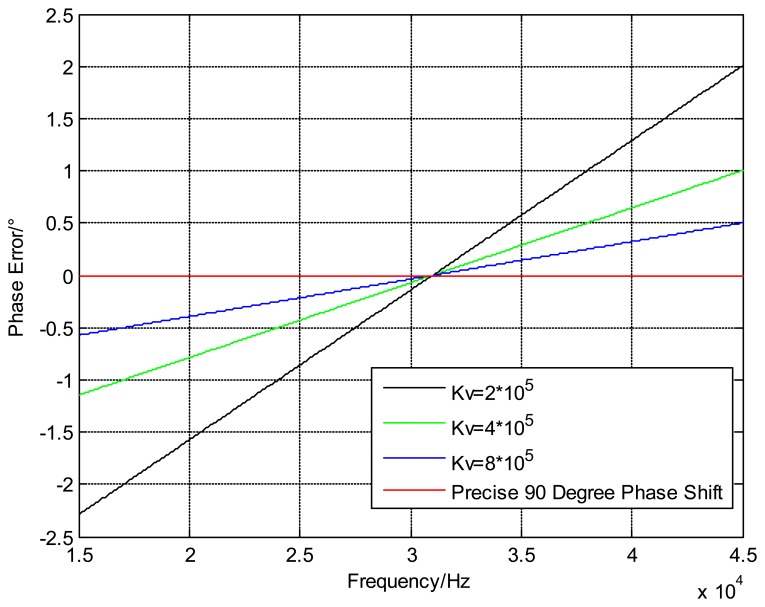
Curves of the variation of the steady-state phase error.

The steady-state phase error can be decreased by changing the gain *K_v_* and the filter's DC gain F(0) in the circuit circle, but this phase error cannot be completely eliminated. The phase error curves of the common passive filter with different *K_v_* values and the precise 90° phase shifter are shown in Figure 13, and it is obvious that the steady-state phase error is basically removed in the 90° phase shifter circuit.

## 4. Fabrication and Packaging

**Figure 14.**
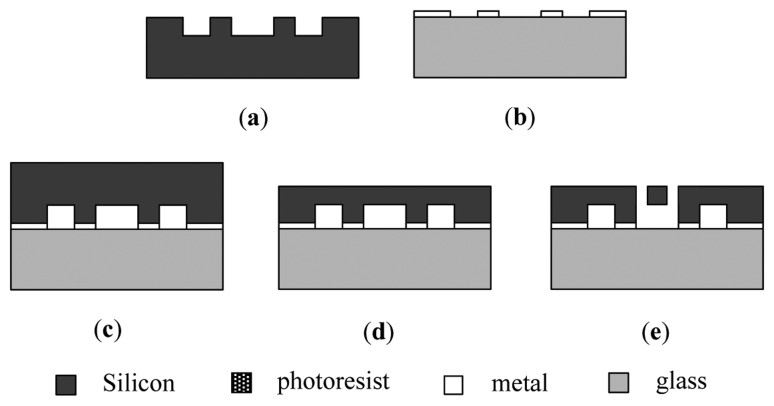
The standard SOG process. (**a**) Etch bonding area on the silicon wafer; (**b**) Depositing the metal electrodes onto the glass substrate; (**c**) Bonding the glass substrate to the silicon wafer; (**d**) Thinning and polishing the silicon wafer; (**e**) DRIE through the silicon wafer to release the structure.

The micromechanical silicon resonant accelerometer is fabricated using the SOG technique. Silicon and glass are the structural layer and the substrate of the MEMS device, respectively. The standard SOG process is illustrated in Figure 14 [24].

**Figure 15.**
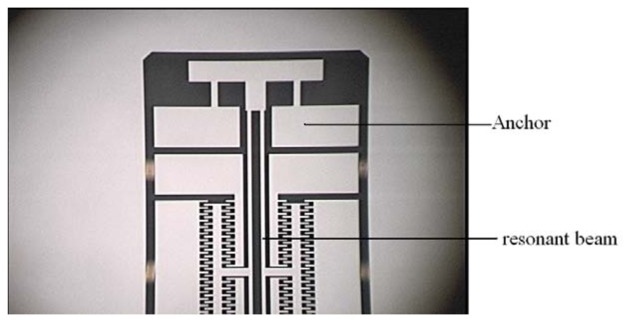
The local structure of the improved micromechanical silicon resonant accelerometer under the 3D video microscope.

**Figure 16.**
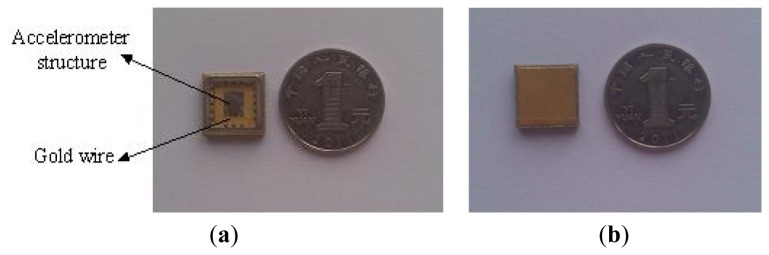
(**a**) The structure after wire bonding; (**b**) The structure after cap sealing.

Figure 15 shows that the local structure of the improved micromechanical silicon resonant accelerometer under the 3D video microscope. The structure of the micromechanical silicon resonant accelerometer is encapsulated using ceramic vacuum packaging. First, the chip mounter is used to fix the structure in the ceramic packing pedestal. Then, the packing is placed into a high-temperature box to solidify the adhesive. The wire bonding is performed using the wire bonder. Finally, vacuum pumping and cap sealing are performed. Figure 16a is the structure after wire bonding, and Figure 16b is the structure after cap sealing.

## 5. Experimental Results and Discussion

**Figure 17.**
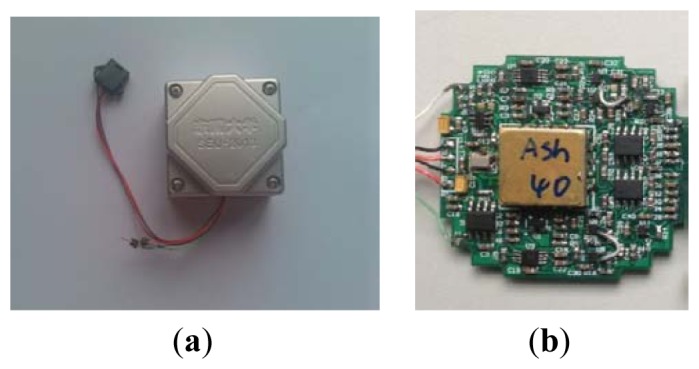
(**a**) The laboratory prototype of the micromechanical silicon resonant accelerometer; (**b**) The circuit module inside the prototype.

Figure 17a is the laboratory prototype of micromechanical silicon resonant accelerometer, and Figure 17b is the circuit module inside the prototype. The prototype is exteriorly connected to two frequency meters to output the test signal. According to the test, at room temperature, the unloaded resonant frequencies of the two resonators are approximately 31.4 kHz and 31.5 kHz, respectively. There is a difference between the two resonators because of the fabrication error and the residual stress. The performance parameters of the prototype were examined by referencing to the domestic testing methods of the resonant accelerometer. The performance tests of the improved accelerometer (shown in subsections 5.1–5.5) were finished on the precision dividing head. The precision of the dividing head is 0.25 arc-second.

### 5.1. Scale Factor

First, the accelerometer is electrically pre-heated at room temperature. There is an input of ±1 *g* into the accelerometer. The output data of the accelerometer is recorded at a sampling frequency of 1 Hz. The measurement time at each point does not exceed 30 s. The measurements are averaged. The scale factor *K*_1_ is calculated according to Equation (21):

(21) $$K_{1}=\frac{U_{+1g}-U_{-1g}}{2}$$

where *U*_+_*_1g_* and *U*_-_*_1g_* are the output of the accelerometer when the acceleration input is +1 g and −1 g, respectively. According to the test result, *U*_+_*_1g_* is 171.1682 Hz, *U*_-_*_1g_* is 38.68814 Hz, and the scale factor of the prototype is 66.24003 Hz/g.

### 5.2. Scale Factor Stability

After the pre-heating at room temperature, 7 scale factors were repeatedly measured for one start-up. The time of each measurement is 10 min. The scale factor stability is calculated using Equation (22):

(22) $$K_{1stab}=\frac{1}{{\bar{K}}_{1}}{\left[\frac{1}{n-1}\overset{n}{\underset{m=1}{\text{∑}}}{\left(K_{1m}-{\bar{K}}_{1}\right)}^{2}\right]}^{\overset{1}{\;}/\underset{2}{\;}}$$

where *K_1stab_* is the scale factor stability, *K_1m_* is the scale factor of the m-th measurement, *K̄*_1_ is the mean of the scale factor, and is the total number of measurements. Table 4 shows the scale factors of the 7 measurements for one start-up. Thus, the scale factor stability of the prototype is calculated as 14.886 ppm.

### 5.3. Scale Factor Repeatability

The accelerometer is powered off for 30 min before the next round of measurements. The scale factor of the accelerometer is measured using the detailed rules in Section 5.1. The accelerometer is powered off six times, *i.e.*, seven measurements are performed. The scale factor repeatability is calculated using Equation (23):

(23) $$K_{1r}=\frac{1}{{\bar{K}}_{1}}{\left[\frac{1}{n-1}\overset{n}{\underset{m=1}{\text{∑}}}{\left(K_{1m}-{\bar{K}}_{1}\right)}^{2}\right]}^{\overset{1}{\;}/\underset{2}{\;}}$$

where *K_1r_* is the scale factor repeatability, and *K_1m_* is the scale factor of the m-th measurement. Table 5 lists the scale factors of the 7 measurements for 7 start-ups. Thus, the calculated scale factor repeatability of the prototype is 23 ppm.

### 5.4. Bias Stability

**Figure 18.**
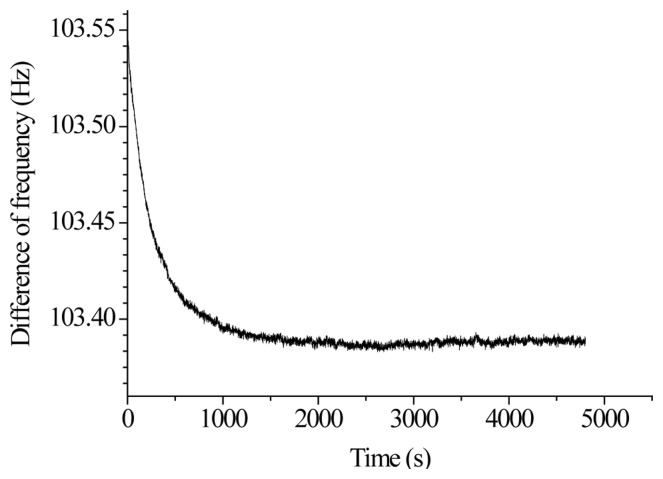
The bias stability measurement curve.

A clamp is installed to ensure that the input shaft of the accelerometer is in the horizontal position, which is nearly 0 g. After the accelerometer is electrically pre-heated for 20 min at room temperature, the prototype is tested for 60 min at a sampling rate of 1 Hz. The average is taken for every 10 groups of 3,600 groups of data. The standard deviation (1 σ) is calculated as the stability index. Figure 18 shows the bias stability measurement curve. The calculated bias stability of the prototype is 23 μg.

### 5.5. Bias Repeatability

After pre-heating at room temperature, the accelerometer is rolled over at four positions, namely +0 g, +1 g, −0 g and −1 g. The output velocity is recorded at a sampling frequency of 1 Hz. The time of each measurement should not exceed 30 s. The measurements are averaged. The bias is calculated using Equation (24):

(24) $$K_{0}=\frac{U_{+0g}+U_{-0g}}{\left(U_{+1g}-U_{-1g}\right)/g}=\frac{U_{+0g}+U_{-0g}}{2K_{1}}$$

where *U*_+_*_0g_* and *U_+0g_* are the outputs of the accelerometer when the acceleration input is +0 g and −0 g, respectively.

The accelerometer is powered off for 30 min at room temperature. Then, it is pre-heated again, and the accelerometer bias is measured. The accelerometer is powered off six times, and seven measurements are conducted. The bias repeatability is calculated using Equation (25) as follows:

(25) $$K_{0r}={\left[\frac{1}{n-1}\overset{n}{\underset{m=1}{\text{∑}}}{\left(K_{0m}-{\bar{K}}_{0}\right)}^{2}\right]}^{\overset{1}{\;}/\underset{2}{\;}}$$

where *K*_0_*_r_* is the bias repeatability, *K*_0_*_m_* is the bias of the m-th measurement, and *K̄*_0_ is the bias mean. Table 6 lists the bias values of seven measurements for seven start-ups. The calculated bias repeatability of the prototype is 170 μg.

### 5.6. Full Temperature Test

The accelerometer is placed in a temperature-controlled oven, where the temperature is maintained at −40 °C, −20 °C, 0 °C, +20 °C, +40 °C and +60 °C, each for 1 h. Then, the resonant frequency of the DETF at each temperature is measured. The output data of the DETF is recorded at a sampling frequency of 1 Hz. The measurement time at each temperature is 30 s. The average value of 30 samples is considered the output at each temperature.

The test results of the two kinds of accelerometers are listed in Tables 7 and 8, respectively. Table 7 shows that the two working frequencies of the improved accelerometer shift by 182.9 Hz and 175.56 Hz, respectively in the temperature range. Table 8 shows that the two working frequencies of the old accelerometer shift by 359.76 Hz and 373.02 Hz, respectively. Although the frequency shifts of two resonators are large, most error is eliminated after the differential output. From −40 °C to +60 °C, the differential output of the improved accelerometer shifts by 7.34 Hz, and the bias temperature coefficient is 0.0734 Hz/°C. The differential output of the old accelerometer shifts by 13.26 Hz, and the bias temperature coefficient is 0.1326 Hz/°C. Obviously the temperature characteristic of the improved accelerometer is superior to the temperature characteristic of the old accelerometer.

## 6. Conclusions

The accelerometer structure is improved to reduce the temperature effect on the accelerometer performance. In practical applications, a different structure can eliminate most of the common-mode errors that are caused by the temperature effects. The improved DETF structure can greatly reduce the thermal stress caused by the temperature change. A complete set of micromechanical silicon resonant accelerometers are designed and fabricated. The closed-loop driving mechanism based on the phase-locked loop is analyzed in detail, and the corresponding circuit diagram is realized. A prototype of the micromechanical silicon resonant accelerometer is developed. According to the test results, the unloaded resonant frequencies of the prototype are approximately 31.4 kHz and 31.5 kHz, respectively; the scale factor is 66.24003 Hz/g; the scale factor stability is 14.886 ppm; the scale factor repeatability is 23 ppm; the bias stability is 23 μg; and the bias repeatability is 170 μg. The bias temperature coefficient is 0.0734 Hz/°C. The test provides the basis for the subsequent development of micromechanical silicon resonant accelerometer prototypes. There is a gap between the prototype and the existing state-of-the-art micromechanical silicon resonant accelerometer. The study on temperature compensation and temperature control is in progress, which can further reduce the temperature effect on the accelerometer and improve the accelerometer performance.

## Figures and Tables

**Table 1.** Resonant frequency of the DETF at different temperatures (reverse motion).

**Temperature (**°**C)**	−**40**	**20**	**60**
Frequency of the common DETF structure (Hz)	28,288.2	31,287.5	33,130.0
Frequency of the improved DETF structure (Hz)	29,439.3	31,282.0	32,449.7

**Table 2.** The parameters of the microleverage mechanism.

**Parameter**	**Length (μm)**	**Width (μm)**	**Depth (μm)**
Microleverage	800	50	60
Input beam	120	8	60
Output beam	50	8	60
Pivot beam	50	8	60

**Table 3.** Working frequencies of the resonators in the two types of accelerometers at different temperatures.

**Temperature (**°**C)**	−**40**	**20**	**60**
Working frequency of the common DETF (Hz)	31,080.9	31,280.5	31,412.7
Working frequency of the improved DETF (Hz)	31,236.2	31,279.8	31,308.8

**Table 4.** The scale factors of the seven measurements for one start-up.

**Number**	**1**	**2**	**3**	**4**	**5**	**6**	**7**
Scale factor (Hz/g)	66.24005	66.24165	66.23948	66.24175	66.24052	66.24018	66.24201

**Table 5.** The scale factors of the seven measurements for seven start-ups.

**Number**	**1**	**2**	**3**	**4**	**5**	**6**	**7**
Scale factor (Hz/g)	66.24730	66.24791	66.25065	66.25112	66.25086	66.25064	66.25027

**Table 6.** The bias values of seven measurements for seven start-ups.

**Number**	**1**	**2**	**3**	**4**	**5**	**6**	**7**
Bias (g)	1.589646	1.589737	1.589785	1.589854	1.589968	1.589993	1.590142

**Table 7.** The full temperature test result of the improved accelerometer.

**Temperature (**°**C)**	−**40**	−**20**	**0**	+**20**	+**40**	+**60**
Resonant frequency of the upper resonator (Hz)	31,331.12	31,379.3	31,426.16	31,462.37	31,496.56	31,514.02
Resonant frequency of the lower resonator (Hz)	31,439.55	31,486.05	31,531.33	31,565.55	31,598.49	31,615.11
Frequency difference (Hz)	108.43	106.75	105.17	103.18	101.93	101.09

**Table 8.** The full temperature test result of the old accelerometer.

**Temperature (**°**C)**	−**40**	−**20**	**0**	+**20**	+**40**	+**60**
Resonant frequency of the upper resonator (Hz)	31,327.09	31,402.31	31,471.82	31,544.49	31,617.52	31,686.85
Resonant frequency of the lower resonator (Hz)	31,402.41	31,479.45	31,551.78	31,627.08	31,702.98	31,775.43
Frequency difference (Hz)	75.32	77.14	79.96	82.59	85.46	88.58
